# The Reciprocal Interactions between Polyphenols and Gut Microbiota and Effects on Bioaccessibility

**DOI:** 10.3390/nu8020078

**Published:** 2016-02-06

**Authors:** Tugba Ozdal, David A. Sela, Jianbo Xiao, Dilek Boyacioglu, Fang Chen, Esra Capanoglu

**Affiliations:** 1 Department of Food Engineering, Faculty of Engineering and Architecture, Okan Univesity, Tuzla, Istanbul TR-34959, Turkey; tugba.ozdal@okan.edu.tr; 2 Department of Food Science, University of Massachusetts Amherst, Amherst, MA 01003, USA; davidsela@umass.edu; 3 Institute of Chinese Medical Sciences, State Key Laboratory of Quality Research in Chinese Medicine, University of Macau, Taipa, Macau, China; jianboxiao@umac.mo; 4 Department of Food Engineering, Faculty of Chemical and Metallurgical Engineering, Istanbul Technical University, Maslak, Istanbul TR-34469, Turkey; boyaci@itu.edu.tr; 5 College of Food Science and Nutritional Engineering, National Engineering Research Centre for Fruits and Vegetables Processing, China Agricultural University, Beijing 100083, China; chenfangch@sina.com

**Keywords:** polyphenols, phenolics, flavonoids, gut microbiota, microbial metabolism, bioavailability, health, interactions

## Abstract

As of late, polyphenols have increasingly interested the scientific community due to their proposed health benefits. Much of this attention has focused on their bioavailability. Polyphenol–gut microbiota interactions should be considered to understand their biological functions. The dichotomy between the biotransformation of polyphenols into their metabolites by gut microbiota and the modulation of gut microbiota composition by polyphenols contributes to positive health outcomes. Although there are many studies on the *in vivo* bioavailability of polyphenols, the mutual relationship between polyphenols and gut microbiota is not fully understood. This review focuses on the biotransformation of polyphenols by gut microbiota, modulation of gut microbiota by polyphenols, and the effects of these two-way mutual interactions on polyphenol bioavailability, and ultimately, human health.

## 1. Introduction

Polyphenols are secondary metabolites found abundantly in a wide variety of foods, such as fruits, vegetables, herbs, seeds and cereals, and in beverages, such as coffee, tea, cocoa and wine [1]. They are currently a topic of great scientific attention due to the interest in their potential health benefits, which include anti-cancer, anti-oxidant, anti-microbial, anti-inflammatory properties. Polyphenols are also implicated in preventing chronic diseases such as cardiovascular diseases, diabetes, obesity, and neurodegenerative diseases, among others [2,3,4,5].

Possible beneficial effects of polyphenols are determined by their bioavailability, among which considerable differences have been observed [6]. Bioavailability is affected by many physicochemical factors such as the type of bioactive compounds, their polarity, molecular mass, plant matrix, their solid state (crystalline *vs.* amorphous), and digestibility by gastrointestinal enzymes, and absorption into the enterocytes. Bioaccessibility, the determinant of release and solubility of bioactive compounds during digestion for further uptake and absorption, is another important factor for bioavailability [7]. In order to understand bioavailability of phenolic compounds and their potential benefits, determination of bioaccessibility during digestion is important.

Most polyphenols pass through the small intestine without being absorbed, thus encountering the gut microbiota which colonizes the colon [8]. This has led to the development of a two-way mutual reaction between polyphenolic compounds and gut microbiota. First, polyphenols are biotransformed into their metabolites by gut microbiota that results in the increased bioavailability of polyphenols. Second, polyphenols modulate the composition of the gut microbial community mostly through the inhibition of pathogenic bacteria and the stimulation of beneficial bacteria. In the latter, they may act as a prebiotic metabolite and enrich the beneficial bacteria [9]. Therefore, the interactions of dietary polyphenols and gut microbiota may result in impact on human host health.

Due to the recent attention paid to these interactions, a number of important reviews have been published focusing on both ends of the relationship—the effect of phenolics on the gut microbiota composition, and the effect of gut microbiota on the biotransformation of phenolic compounds, their bioavailability, or human health [10,11,12,13,14,15,16,17,18,19]. The aim of this review is to provide an overview of the two-way reciprocal relationship of all the sub-classes of phenolic compounds and gut microbiota and the relevance of these interactions to bioavailability and human health.

## 2. Polyphenols, Gut Microbiota and Health

Polyphenols are a large group of heterogeneous compounds characterized by hydroxylated phenyl moieties, and are found mostly in plants, including fruits, vegetables, and cereals, as well as derived beverages such as tea, coffee and wine [20]. Polyphenols have become an intense focus of research due to their potential benefits to health, particularly in relation to the prevention of cancer [21,22] and cardiovascular diseases [23,24]. Suggested beneficial effects are anticarcinogenic [25,26], antiatherogenic [27,28], antiulcer [29], antithrombotic [30,31], anti-inflammatory [32,33], antiallergenic [34,35], anticoagulant [36], immune modulating [37], antimicrobial [38,39], vasodilatory [40], and analgesic activities [41]. To achieve these health benefits, polyphenols require *in situ* processing by the gut microbiota to be transformed into a potentially more bioactive, low-molecular-weight metabolite [42]. Faria *et al.* (2014) reviewed that total polyphenol absorption in the small intestine is relatively low (5%–10%) in comparison to other macro- or micronutrients. The remaining 90%–95% of polyphenols transit to the large intestinal lumen and accumulate in the millimolar range. From the lumen, together with conjugates excreted from bile, they are exposed to the enzymatic activities of the gut microbiota [43].

The microbiota that colonize the distal regions of the colon represent the highest concentration of microorganisms found in human body, as well as the most diverse [44]. It is known that the human gut has an ecosystem of around 10^13^–10^14^ bacterial cells, an estimate 10 times that of human somatic cells [45]. In addition, the aggregate microbial genome (*i.e.*, microbiome) is predicted to contain more than three million genes, or 150 times more than human genes [46]. In short, the gut microbiota is essential for the maintenance of intestinal homeostasis and human health [47].

The reciprocal relationship between polyphenols and gut microbiota may contribute to host health benefits. The need to clarify the molecular mechanisms underlying the observed prebiotic enrichment of beneficial bacteria and antimicrobial activities against gut pathogenic bacteria is apparent [42,48,49,50,51,52]. Commensals residing in the gut may improve health by protecting against gastrointestinal disorders and pathogens, processing nutrients, reducing serum cholesterol, strengthening intestinal epithelial tight cell junctions, increasing mucus secretion and modulating intestinal immune response through cytokine stimulus [53,54]. Furthermore, the gut microbiota biotransforms polyphenols into metabolites that may have greater biological activity than their precursor structures [42].

## 3. Metabolism of Phenolics and Microbial/Colonic Metabolic Pathways

**Figure 1:**
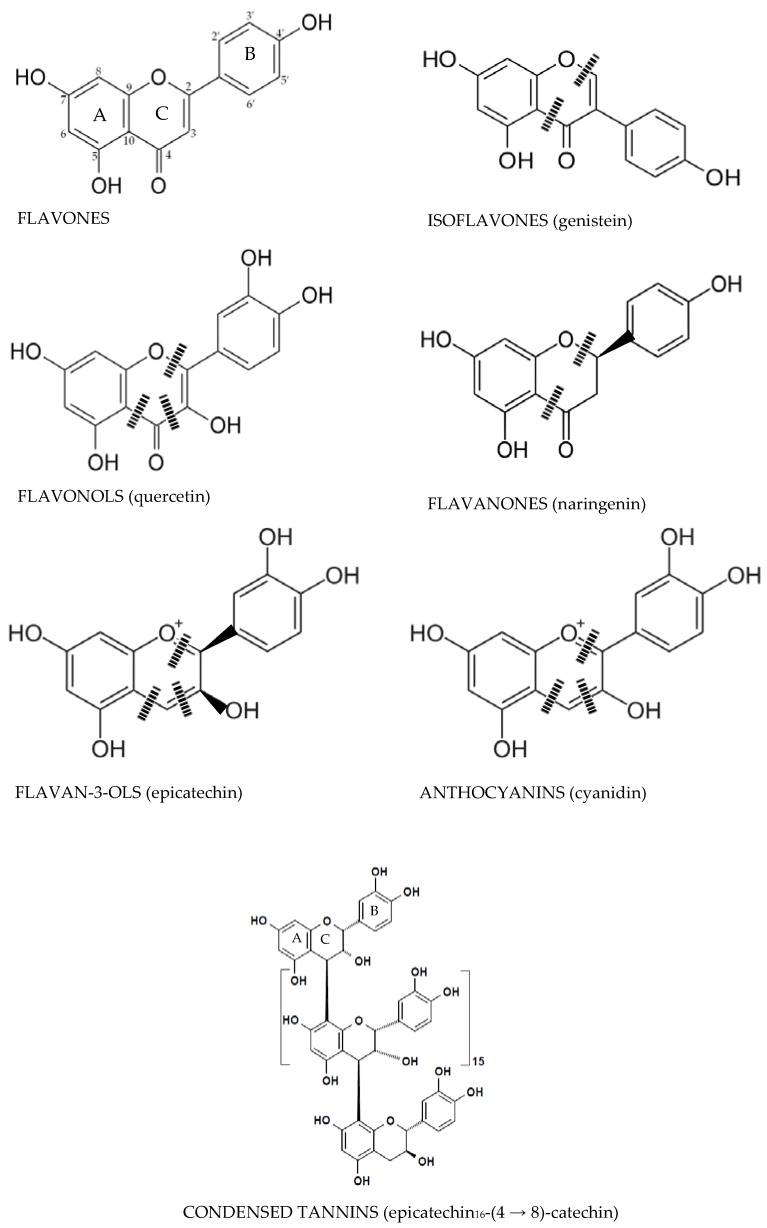
Microbiota heterocyclic C ring cleavage of flavonoids; (|||) positions of the potential C-ring cleavages [42].

**Figure 2:**
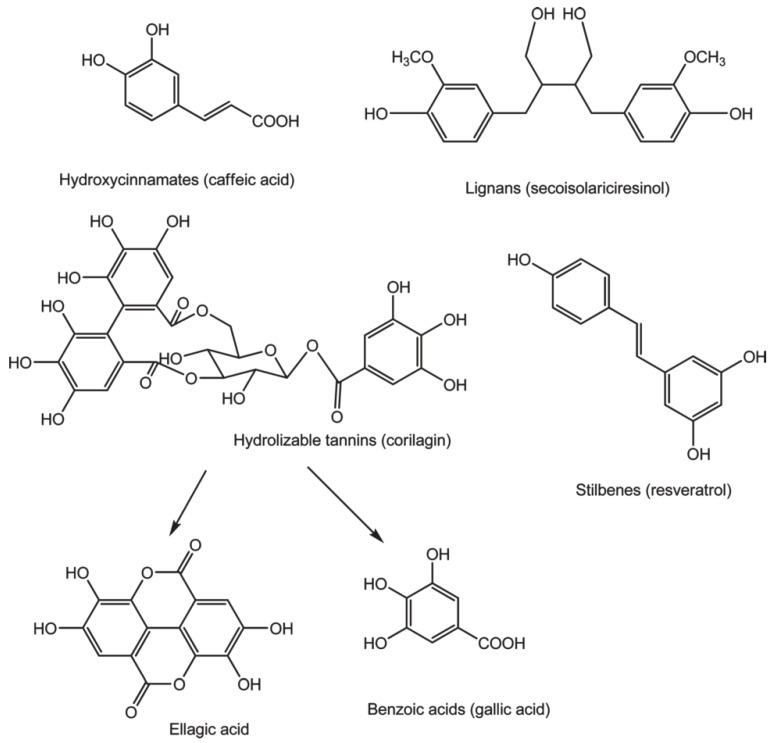
Nonflavonoid-type phenolics that are metabolized by the gut microbiota [42].

Phenolics are mostly found bound to sugars and organic acids that can be grouped into flavonoids and non-flavonoids. Figure 1 and Figure 2 exhibit the structure of flavonoid-type phenolics and nonflavonoid-types, respectively. Flavonoid-type phenolics have a primary structure existing of two benzene rings, A and B, connected through a heterogeneous pyrone C ring. In contrast, nonflavonoid-type phenolics have a more diverse group of compounds from the simplest C6-C1 benzoic acids to more complicated C6-C2-C6 stilbenes, C6-C3-C3-C6 lignans and gallotannins, hydrolyzable tannins and ellagitannins [42].

### 3.1. Flavonoid-Type Phenolics

#### 3.1.1. Flavonols

Flavonols contain a 3-hydroxyflavone base (3-hydroxy-2-phenylchromen-4-one) and have a planar ring system (Figure 1). They are differentiated by their hydroxy modification at distinct positions of the phenol residue. Foods particularly enriched in flavonols are onions, broccoli, tea, apples, and red wine [55]. Flavonols are extensively hydrolyzed into their metabolite-derivative products by gut microbiota at the A and B rings as a result of the C ring cleavage [56,57]. Accordingly, quercetin provides 2-(3,4-dihydroxyphenyl)acetic acid, 2-(3-hydroxyphenyl)acetic acid, and 3,4-dihydroxybenzoic acid from the B ring, while phloroglucinol, 3-(3,4-dihydroxyphenyl)propionic acid, and 3-(3-hydroxyphenyl)propionic acid are formed from the A ring. Additional phenolic-metabolites such as 3-methoxy-4-hydroxy-benzoic acid (vanillic acid), 2,4,6-trihydroxybenzoic acid, 2-(3,4-dihydroxyphenyl)ethanol, 3,4-dihydroxybenzaldehyde, 3-(3,4-dihydroxyphenyl)benzoic acid methyl ester, and 3-(*m* or *p*-hydroxyphenyl) propionic acid have also been recognized. The types of phenolic compounds produced are affected by the mechanism of B ring hydroxylation. Myricetin trihydroxylation produces 2-(3,5-dihydroxyphenyl)acetic acid, 2-(3-hydroxyphenyl)acetic acid, and 2-(3,4,5-trihydroxyphenyl)acetic acid. Moreover, kaempferol (which has a 4′-hydroxy-ring B), releases only 2-(4-hydroxyphenyl)acetic acid. In summary, flavonols are biotransformed by C ring fission, and later by dehydroxylation reactions in the intestine [58].

#### 3.1.2. Flavones and Flavanones

Flavones are flavonoids that have a basic structure consisting of a 2-phenyl-benzo-γ-pyrone skeleton formed by two phenyl rings (A and B) linked with a heterocyclic pyrone ring (C) (Figure 1). Flavanones have a 2,3-dihydro-2-phenylchromen-4-one structure. (Figure 1). The pyran rings of flavanones are nonlinear because they contain saturated C2–C3 bonds. They can form linkages with estrogen receptors. Furthermore, they do not contain double bonds between C2 andC3, contrary to isoflavones (Figure 1). Citrus fruits such as lemon, grapefruit, and orange are the most important dietary sources of flavanones [59].

In comparison, flavanones have a higher bioavailability compared to flavonols and flavan-3-ols. This could be explained in part by less degradation by gut microbiota and greater accessibility for absorption in the intestine. Flavanones occur as glycosides, usually rutinosides (6-*O*-α-l-rhamnosyl-d-glucosides) and neohesperidosides (2-*O*-α-l-rhamnosyl-d-glucosides) at the seventh position [60]. The degradation pathways of flavanone glycosides such as naringin are similar to flavonols. The first reaction is a deglycosylation to form naringenin, which then turns into phloroglucinol and 3-(3,4-dihydroxyphenyl)propionic acid by cleavage of the C ring [58].

#### 3.1.3. Flavone *C*-Glycosides

**Figure 3:**
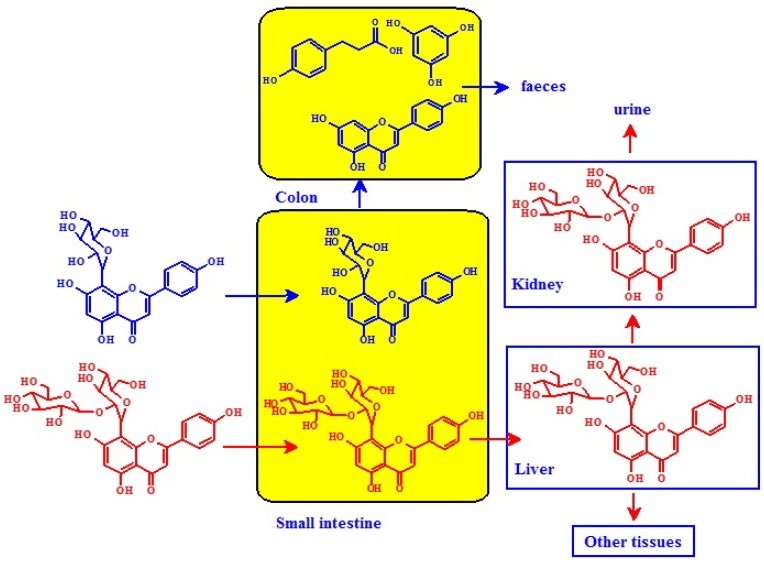
Absorption and metabolism of flavone *C*-monoglycosides (blue arrow) and *C*-multiglycosides (red arrow) [63].

Most dietary flavonoids exist in their *O*-glycosidic forms. However, *C*-glycosylated flavonoids, especially flavones, are widespread in a variety of plants [61]. In most cases, flavone *O*-glycosides are hydrolyzed by digestive enzymes or degraded by gut bacteria to their aglycones in the intestine to be reduced and conjugated to form *O*-glucuronides and *O*-sulfates in the liver [62,63]. However, flavone *C*-monoglucosides exhibit different metabolism pathways compared to flavone *C*-multiglycosides [63] (Figure 3). Flavone *C*-monoglucosides such as orientin, vitexin, homoorientin, and isovitexin are observed to be poorly absorbed in the gastrointestinal tract of rats and, consequently, were able to reach the colon [64]. Once in the colon, flavone *C*-monoglucosides are deglycosylated and degraded to smaller metabolites, such as phloroglucinol, hydrocaffeic acid, and phloretic acid, by human gut bacteria, yielding very few metabolites in the urine and blood. Flavone *C*-multiglycosides are absorbed intact in the intestine and are minimally changed in the liver. Afterwards, they are returned to the gut by enterohepatic recirculation [63].

#### 3.1.4. Isoflavones

Isoflavones have a planar basic ring system with the benzenoid B ring attached to C3, differentiating it from the other flavonoids. Isoflavonoids are nonsteroidal and estrogen-like due to their chemical structure. As they are able to bind to estrogen receptors (ERα and ERβ), they are classified as natural selective estrogen receptor modulators (SERMs) [65,66,67,68,69,70]. Isoflavones are in the form of water-soluble glycosides and they cannot be absorbed completely through the enterocyte. This is a consequence of their high hydrophilicity and molecular weight. Soy is one of the food products rich in health-promoting isoflavonoids [65]. Soy products contain 12 identified isoflovone compounds: three aglycones (genistein, daidzein, glycitein); three glucosides; three acetyl ester glucosides; and three malonyl ester glucosides. Isoflavones can be biotransformed by β-glucosidase from gut microbiota into their aglycones for increased bioavailability [70,71]. The aglycones can be both absorbed completely or metabolized by gut microbiota [72]. Aglycones are also biotransformed into their metabolites, namely daidzein to *O*-demethylangolensin (*O*-DMA) and equol, and genistein to *p*-ethylphenol and 4-hydroxyphenyl-2-propionic acid. Equol has higher antioxidant activity than soy isoflavones due to its nonplanar structure which is more flexible to conformational modification. Though, due to the absence of specific constituents of the gut microbiota, only about one-third of the human population can metabolize daidzein into equol. Other observed microbial metabolites of daidzein are 2-dehydro-*O*-DMA, dihydrodaidzein, tetrahydrodaidzein, 6-hydroxydaidzein, 8-hydroxydaidzein, and 3′-hydroxydaidzein, and 3-(4-hydroxyphenyl)benzopyran-4,7-diol. They are biotransformed by deglucosylation, reduction, C ring fission, and hydroxylation reactions [65]. Soybean products contain less than 10% glycitein, but it comprises about half of the isoflavone mass in soy germ. It is demethoxylated into 6,7,4′-trihydroxyisoflavone *in vitro* by *Eubacterium limosum* [73]. Metabolites of glycitein have been isolated and characterized as dihydroglycitein, 6-*O*-methyl-equol, 5′-*O*-methyl-*O*-desmethylangolensin, and dihydro-6,7,4′-trihydroxyisoflavone [74,75].

#### 3.1.5. Flavanols

Simple flavanols such as (+)-catechin (C), (−)-epicatechin (EC), epigallocatechin, their gallate esters, and polymeric procyanidins from dimers and polymers collectively named condensed tannins are all classified as flavanols. They are the primary contributors to dietary phenolics and are found mostly in fruits, tea, wine and chocolate. Similar to flavanones and flavonols, flavanols such as catechin, epigallocatechin and epicatechin have a B ring attached to C2, but they do not have a carbonyl group in their C4 position or double bonds between C2 and C3. They are not found in a planar conformation as flavanones are [42].

Flavanols are promptly metabolized to several *O*-sulfated, *O*-glucuronidated, and *O*-methylated forms by phase II enzymes [76,77,78,79]. The main phenolic-metabolites of catechin and epicatechin are 5-(3′,4′-dihydroxyphenyl)-γ-valerolactone, 3-(3-hydroxyphenyl)propionic acid, and 3-hydroxyhippuric acid, 5-(3′-hydroxyphenyl)-γ-valerolactone [58,80,81]. Tzounis *et al.* (2008) found out that when (−)-epicatechin or (+)-catechin were incubated with intestinal bacteria, 5-(3′,4′-dihydroxyphenyl)-γ-valerolactone, 5-phenyl-γ-valerolactone, and 3-phenylpropionic acid were formed [48]. For the biotransformation of these metabolites to proceed, initial conversion to (+)-epicatechin from (+)-catechin is essential. The metabolites pyrogallol, 3-(3-hydroxyphenyl)propionic acid, 5-(3,4-dihydroxyphenyl)valeric acid, 5-(3-hydroxyphenyl)valeric acid, 3-(3,4-dihydroxyphenyl)propionic acid, 3-(3-methoxyphenyl) valeric acid, and 2,3-dihydroxyphenoxyl 3-(3′,4′-dihydroxyphenyl)propionic acid are obtained after *in vitro* incubation of epicatechin with the human intestinal microbial community. Dietary condensed tannins are biotransformed into benzoic, phenylacetic, phenylpropionic, and phenyllactic acid derivatives, with phloroglucinol, 5-(3′-hydroxyphenyl)-γ-valerolactone, and 1-(3-hydroxyphenyl)-3-(2,4,6-trihydroxyphenyl)propan-2-ol being produced [82]. In another study, condensed tannins produced 3-(4-hydroxyphenyl)propionic acid, 3-phenylpropionic acid, 2-(3-hydroxyphenyl)acetic acid, 2-(4-hydroxyphenyl)acetic acid, 5-(3-hydroxyphenyl)valeric acid, and 3-(3-hydroxyphenyl)propionic acid by way of the gut microbiota [80].

The dry weight of green tea consists of 30%–42% catechin. Major catechins are (+)-epigallocatechin gallate (EGCG), (−)-epigallocatechin (EGC), (−)-epicatechin gallate (ECG), and (−)-epicatechin (EC), in which EGCG is the most abundant, and may account for 50%–80% of the total catechins in tea [83]. The metabolism of tea catechins by gut microbiota has been studied extensively, demonstrating that the absorption of catechins in the small intestine is relatively low. As a result, the majority of tea catechins are biotransformed by gut microbiota, followed by absorption in the bloodstream or excretion in the feces.

Recently, Takagaki and Nanjo (2013) identified several new metabolites of (+)-C or (−)-EC biotransformed by rat intestinal microbiota and they revised the suggested metabolic pathway of these compounds [84]. They have observed four different strains of human intestinal bacteria that have the ability to biotransform (+)-C and (−)-EC.

Wang *et al.* (2001) reported that *Eubacterium* sp. strain SDG-2 biotransformed (+)-C to 1-(3′,4′-dihydroxyphenyl)-3-(2″,4″,6″-trihydroxyphenyl)propan-2-ol. However, the bacteria biotransformed (−)-EC into two compounds, 1-(3′,4′-dihydroxyphenyl)-3-(2″,4″,6″-trihydroxyphenyl)propan-2-ol and 1-(3′-hydroxyphenyl)-3-(2″,4″,6″-trihydroxyphenyl)propan-2-ol. Thus, *Eubacterium* sp. strain SDG-2 has the ability of *p*-dehydroxylation in the B ring of (−)-EC but not in (+)-C [85].

Recently, Kutschera *et al.* (2011) revealed that both (+)-C and (−)-EC could be biotransformed to 1-(3′,4′-dihydroxyphenyl)-3-(2″,4″,6″-trihydroxyphenyl)propan-2-ol by *Eggerthella lenta* rK3. However, the conversion of (+)-C progressed five times faster than that of (−)-EC. *Flavonifractor plautii* aK2 further converted 1-(3′,4′-dihydroxyphenyl)-3-(2″,4″,6″-trihydroxyphenyl)propan-2-ol to δ-(3′,4′-dihydroxyphenyl)-γ-valerolactone and δ-(3′,4′-dihydroxyphenyl)-γ-valeric acid [86].

EGC can be biotransformed by *Eubacterium* sp. strain SDG-2 into 1-(3′,5′-dihydroxyphenyl)-3-(2″,4″,6″-trihydroxyphenyl)propan-2-ol [85]. Moreover, it was observed that 5-(3′,4′,5′-trihydroxyphenyl)-γ-valerolactone can be formed as an additional major metabolite of EGC [77,78].

Furthermore, it was reported that some phenolic acids were produced such as 3,4-DHPPA, 3-HPPA, 4-HPPA), 3,4-DHPAA, 3-HPAA, 3-HBA, 4-HBA and phloroglucinol [87,88,89]. Meng *et al.* (2002) identified δ-(3′,4′,5′-trihydroxyphenyl)-γ-valerolactone, δ-(3′,4′-dihydroxyphenyl)-γ-valerolactone, and δ-(3′,5′-dihydroxyphenyl)-γ-valerolactone in the urine after EGC consumption [90]. Upon consumption of 200 mg of pure ECG, δ-(3′,4′,5′-trihydroxyphenyl)-γ-valerolactone, δ-(3′,4′-dihydroxyphenyl)-γ-valerolactone, and δ-(3′,5′-dihydroxyphenyl)-γ-valerolactone were identified in the urine [90].

Furthermore, (−)-EGCG is the 3-*O*-gallate product of (−)-EGC, which is the major catechin derivative found in tea. Van’t Slot and Humpf (2009) reported that (+)-GCG and (−)-EGCG were degraded by the intestinal microbiota of pig cecum, and biotransformed into (+)-GC and (−)-EGC, respectively [89]. This degradation is also observed by human and rat intestinal microbiota in *in vitro* models of the colon [88,91]. In addition, Takagaki and Nanjo (2010) analyzed the metabolism and biotransformation of (−)-EGCG by rat intestinal bacteria and proposed three metabolic pathways. In the first step, (−)-EGCG is hydrolyzed into (−)-EGC and gallic acid. Then, EGC is biotransformed into 1-(3′,4′,5′-trihydroxyphenyl)-3-(2″,4″,6″-trihydroxyphenyl)propan-2-ol by reductive opening among the first and second positions of EGC. Subsequently, 1-(3′,4′,5′-trihydroxyphenyl)-3-(2″,4″,6″-trihydroxyphenyl)propan-2-ol is biotransformed into 1-(3′,5′-dihydroxyphenyl)-3-(2″,4″,6″-trihydroxyphenyl)propan-2-ol by dehydroxylation of 1-(3′,4′,5′-trihydroxyphenyl)-3-(2″,4″,6″-trihydroxyphenyl)propan-2-ol at the 4′ position. Moreover, as a moor degradation pathway of EGCG metabolism, 5-(3′,5′-dihydroxyphenyl)-γ-valeric acid is formed as the main metabolite by ring fission of the phloroglucinol moiety of the metabolite 1-(3′,5′-dihydroxyphenyl)-3-(2″,4″,6″-trihydroxyphenyl)propan-2-ol. This is the major pathway of EGCG metabolism. Simultaneously, just after the ring fission, the 5-(3′,5′-dihydroxyphenyl)-γ-valerolactone metabolite may be formed according to the lactonization of 5-(3′,5′-dihydroxyphenyl)-γ-valeric acid and its small part is biotransformed into 3,5-dihydroxyphenyl-propionic acid. In addition, an insignificant amount of 4′-dehydroxylated metabolite is biotransformed from EGC that is not further metabolized by the gut microbiota [91].

There are also *in vivo* studies on the metabolism of EGCG explaining the likely metabolic pathway as in *in vitro* studies. *In vivo* studies on the biotransformation of EGCG after oral administration to rats revealed that EGCG was relocated into the cecum and large intestine, and then underwent degradation by intestinal bacteria to 5-(3′,5′-dihydroxyphenyl)- γ-valerolactone with EGC as an intermediate product [92,93]. An abundant quantity of 5-(3′,5′-dihydroxyphenyl)-γ-valerolactone is absorbed in the body by going through glucuronidation in the intestinal mucosa or liver, transformed to its glucuronidated metabolites which enter blood circulation. Next they are distributed to various tissues and excreted in the urine [90,92,93]. *In vivo* and *in vitro* biotransformations of flavan-3-ols are given in detail with their degradation products in Table 1.

**Table 1:** *In vitro* and *in vivo* biotransformation of flavan-3-ols by gut microbiota.

Compound	Metabolite	Model (*in Vivo*/*in Vitro*) and References
(+)-C or (−)-EC	1-(4′-hydroxyphenyl)-3-(2″,4″,6″- trihydroxyphenyl)propan-2-ol	Rat *in vitro* [84]
	1-(3′-hydroxyphenyl)-3-(2″,4″,6″- trihydroxyphenyl)propan-2-ol	Rat *in vitro* [84]
	1-(3′,4′-dihydroxyphenyl)-3-(2″,4″,6″-trihydroxyphenyl)propan-2-ol	Rat *in vitro* [84]
	5-(3′-hydroxyphenyl)pentanoic acid	Rat *in vitro* [84]
	5-(3′,4′-dihydroxyphenyl)-4-oxo-valeric acid	Rat *in vitro* [84]
	5-(3′-hydroxyphenyl)-4-oxo-valeric acid	Rat *in vitro* [84]
	5-[(3′,4′-dihydroxyphenyl)methyl]oxolan-2-one	Rat *in vitro* [84]; Human *in vitro* [87]; Human *in vivo* [90]
	5-[(3′-hydroxyphenyl)methyl)oxolan-2-one	Rat *in vitro* [84]; Human *in vitro* [88]
	5-(3′,4′-dihydroxyphenyl)-pentanoic acid	Rat *in vitro* [84]; Human *in vitro* [87]
	3,4-DHPPA	Rat *in vitro* [84]; Pig *in vitro* [89]
	3-HPPA	Rat *in vitro* [84]; Human *in vitro* [87]
	4-HPAA	Pig *in vitro* [89]
	3-HBA	Pig *in vitro* [89]
	4-HBA	Pig *in vitro* [89]
	Phloroglucinol	Pig *in vitro* [89]
	5-[(3′,4′,5′-trihydroxyphenyl)methyl]oxolan-2-one	Human *in vitro* [88]
(+)-GC or (–)EGC	1-(3′,5′-dihydroxyphenyl)-3-(2″,4″,6″- trihydroxyphenyl)propan-2-ol	Human *in vitro* [87,88]
	5-[(3′,4′,5′-trihydroxyphenyl)methyl]oxolan-2-one	Human *in vitro* [87,88]
	4-HPAA	Human *in vitro* [87]; Pig *in vitro* [89]
	Phloroglucinol	Pig *in vitro* [89]
	3,4-DHPPA	Pig *in vitro* [89]
	3-HPPA	Pig *in vitro* [89]
	3-HBA	Pig *in vitro* [89]
	4-HBA	Pig *in vitro* [89]
(−)-EGC	5-[(3′,4′,5′-trihydroxyphenyl)methyl]oxolan-2-one	Human *in vivo* [90]
	5-[(3′,4′-dihydroxyphenyl)methyl]oxolan-2-one	Human *in vivo* [90]
	5-[(3′,5′-dihydroxyphenyl)methyl)]oxolan-2-one	Human *in vivo* [90]
(−)-ECG	EC	Rat *in vivo* [92,93]
	Gallic acid	Rat *in vivo* [92,93]
	Pyrogallol	Rat *in vivo* [92,93]
	1-(3′,4′-dihydroxyphenyl)-3-(2″,4″,6″- trihydroxyphenyl)propan-2-ol	Rat *in vivo* [92,93]
	5-[(3′,4′-dihydroxyphenyl)methyl]oxolan-2-one	Rat *in vivo* [92,93]
	5-[(3′-hydroxyphenyl)methyl)]oxolan-2-one	Rat *in vivo* [92,93]
	5-(3′,4′-dihydroxyphenyl)pentanoic acid	Rat *in vivo* [92,93]
	3-HPPA	Rat *in vivo* [92,93]
	(E)-3-(3-hydroxyphenyl)-acrylic acid	Rat *in vivo* [92,93]
	EGC	Rat *in vivo* [92,93]
(+)-GCG or (−)-EGCG	EGC	Rat *in vitro* [91]; Human *in vitro* [87,88]; Pig *in vitro* [89]
	Gallic acid	Rat *in vitro* [91]; Human *in vitro* [87,88]; Pig *in vitro* [89]
	5-[(3′,4′,5′-trihydroxyphenyl)methyl]oxolan-2-one	Rat *in vitro* [91]; Pig *in vitro* [89]
	1-(3′,4′,5′-trihydroxyphenyl)-3-(2″,4″,6″-trihydroxyphenyl)propan-2-ol	Rat *in vitro* [91]
	1-(3′,5′-dihydroxyphenyl)-3-(2″,4″,6″- trihydroxyphenyl)propan-2-ol	Rat *in vitro* [91]
	5-(3′,5′,- dihydroxyphenyl) pentanoic acid	Rat *in vitro* [91]
	5-(3′,4′,5′-trihydroxyphenyl) pentanoic acid	Rat *in vitro* [91]
	5-(3′-hydroxyphenyl)-pentanoic acid	Rat *in vitro* [91]
	5-[(3′,5′-dihydroxyphenyl)methyl)]oxolan-2-one	Rat *in vitro* [91]
	3,5-DHPPA	Rat *in vitro* [91]
	5-[(3′,4′,5′-trihydroxyphenyl)methyl]oxolan-2-one	Human *in vitro* [87]
	Pyrogallol	Human *in vitro* [87]
	Pyrocatechol	Human *in vitro* [87]
	4-HPAA	Human *in vitro* [87]
(−)-EGCG	EGC	Rat *in vivo* [92,93]
	Gallic acid	Rat *in vivo* [92,93]
	1-(3′,4′,5′-trihydroxyphenyl)-3-(2″,4″,6″- trihydroxyphenyl)propan-2-ol	Rat *in vivo* [92,93]
	1-(3′,5′-dihydroxyphenyl)-3-(2″,4″,6″-trihydroxyphenyl)propan-2-ol	Rat *in vivo* [92,93]
	5-[(3′,4′,5′-trihydroxyphenyl)methyl]oxolan-2-one	Rat *in vivo* [92,93]
	5-[(3′,5′-dihydroxyphenyl)methyl)]oxolan-2-one	Rat *in vivo* [92,93]
	5-[(3′,4′-dihydroxyphenyl)methyl]oxolan-2-one	Rat *in vivo* [92,93]

Abbreviations: 3,4-DHPPA, 3,4-dihydroxyphenylpropionic acid; 3-HPPA, 3-hydroxyphenylpropionic acid; 4-HPPA, 4-hydroxyphenylpropionic acid; 3-HBA, 3-hydroxybenzoic acid; 4-HBA, 4-hydroxybenzoic acid; 3,4-DHPAA, 3,4-dihydroxyphenylacetic acid; 3-HPAA, 3-hydroxyphenylacetic acid; 4-HPAA, 4-hydroxyphenylacetic acid.

#### 3.1.6. Anthocyanins

Anthocyanins belong to a large group of secondary plant metabolites collectively known as flavonoids [94]. They are responsible for the red and blue pigmentation of many fruits and vegetables [95]. The health benefits of anthocyanins have been demonstrated in several *in vivo* and *in vitro* studies [96,97,98]. However, the low bioavailability of anthocyanins is a clear obstacle in achieving desired beneficial effects [99].

Several studies on the intestinal absorption of anthocyanins have been reported. The anthocyanins delphinidin 3-*O*-rutinoside, cyanidin 3-*O*-rutinoside, delphinidin 3-*O*-glucoside, and cyanidin 3-*O*-glucoside were found to be directly absorbed and excreted as the glycosylated forms in both rats and human subjects [100]. A number of studies showed that the proportion of total anthocyanins absorbed and subsequently excreted in urine was far below 1% [101]. By collecting ileostomy effluent after the consumption of anthocyanin-rich blueberries, Kahle *et al.* (2006) determined that up to 85% of blueberry anthocyanins reach the colon under physiological conditions [102]. However, about 69% of the anthocyanins disappeared from the gastrointestinal tract within 4 h after food ingestion [103,104]. As demonstrated by *in vitro* fermentation of anthocyanins seeded by a fecal community obtained from rats, cyanidin-3-glucoside and cyanidin-3-rutinoside extracted from wild mulberry were completely degraded within 10 hours [105]. It is thus likely that intestinal microbiota contribute to the biotransformation and the metabolism of anthocyanins [106].

**Figure 4:**
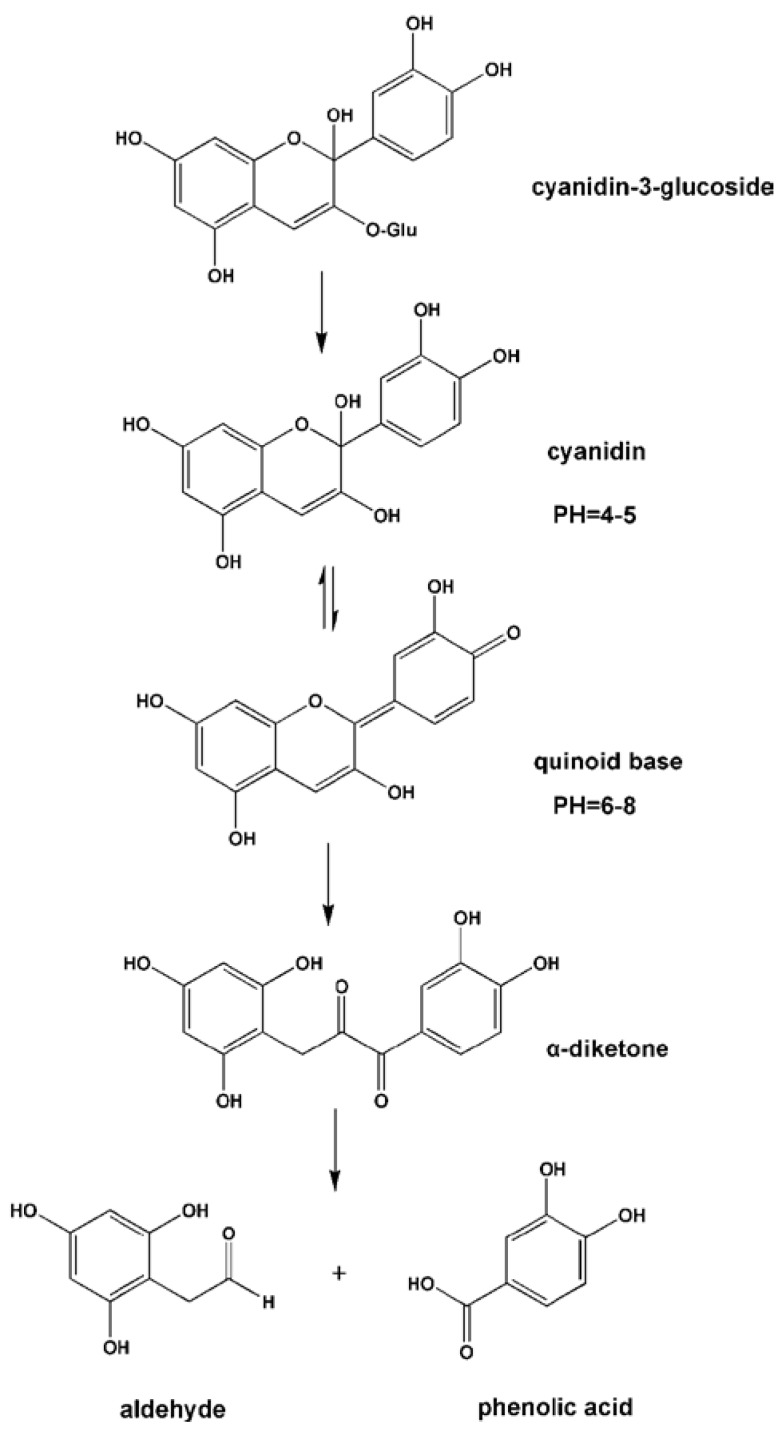
pH-dependent structural changes and degradation of cyanidin-3-glucoside. Reproduced from the original source [94].

Fleschhut *et al.* (2006) incubated an anthocyanin-rich extract from red radish with human fecal suspension *in vitro* and demonstrated that the first step of the bacterial hydrolysis of anthocyanins (*i.e.*, monoglucosides, diglucosides as well as acylated anthocyanins) involves the cleavage of the sugar moiety leading to the formation of the anthocyanin aglycon. The activities of two bacterial enzymes in particular, α,l-rhamnosidase and β,d-glucosidase, may be responsible for the deglycosylation of anthocyanins [94]. Due to the high instability of the liberated aglycones at the pH of various locations in the intestine, they might spontaneously change to form quinoid bases, which further break down into a phenolic acid and an aldehyde via an α-diketone intermediate (Figure 4). Therefore, the major degradatory pathway of this process is the formation of the phenolic acid descending from the B ring of the anthocyanin skeleton (Table 2) [94,95].

Many studies have shown that protocatechuic acid is one of the most likely major degradation products of anthocyanins. In a human study, Vitaglione *et al.* (2007) identified that protocatechuic acid was the major metabolite of cyanidin-*O*-glucosides, accounting for almost 73% of ingested cyanidin-*O*-glucosides [101]. After consumption of black raspberries in a porcine model, the phenolic acid profile in the gastrointestinal tract indicated that protocatechuic acid was the major metabolic derivative, followed by *p*-coumaric acid, caffeic acid, ferulic acid and 3-hydroxybenzoic acid [107]. In addition, a high level of protocatechuic acid was also identified in rat plasma after the oral intake of cyanidin 3-*O*-β-d-glucoside [108]. Cyanidin 3-*O*-β-d-glucoside was reported to metabolize to protocatechuic acid *via* cyanidine when incubated with fecal microbiota in an *in vitro* model. This resulted in a more potent anti-scratching behavioral effect than the parent cyanidin 3-*O*-β-d-glucoside in mice, thus suggesting the biological activity of anthocyanins *in vivo* may increase due to their metabolites, such as phenolic acids [109].

**Table 2:** The expected B ring fragments for the common anthocyanidins. Reproduced from the original source [110].

Anthocyanidin	Initial B-Ring Fragmentation Product
Pelargonidin	4-Hydroxybenzoic acid
Cyanidin	Protocatechuic acid
Delphinidin	Gallic acid
Peonidin	Vanillic acid
Petunidin	3-Methoxy-4,5-dihydroxybenzoic acid
Malvidin	Syringic acid

### 3.2. Nonflavonoid-Type Phenolics

#### 3.2.1. Phenolic Acids

Phenolic acids are found in many foods, and in high concentrations in whole grains, wine and berries. Cereals contain mostly the hydroxycinnamate ferulic acid as a phenolic acid. It is found bound to arabinoxylans by ester bonds in rye. Therefore, esterases (e.g., cinnamoyl estarase) are required during digestion or by the gut microbiota to improve its bioaccessibility [111,112]. As a result of this hydrolysis, unbound phenolic acids can be absorbed through the gastrointestinal barrier and enter peripheral circulation [113].

Moreover, *in vitro* hydroxycinnamic acid conversions by gut microbiota can be observed. In a study conducted by Gonthier and colleagues (2006), caffeic acid and its esters, caftaric acid and chlorogenic acid, were used as a substrate in a human colon model and phenolic acids were transformed into 3-(3′-hydroxyphenyl)propionic acid and minor quantities of benzoic acid. The side-chain shortening of phenylpropionic acid arises through β-oxidation [114]. Caffeic acid can also be decarboxylated and metabolized into 4-ethylcatechol by gut microbiota [115].

Ferulic acid dimers are other important phenolic acids released from cereals [116]. Two ferulic acids can be bound to each other either through 8-*O*-4- or 5-5-linkages. In the study of Braune *et al.* (2009), dehydrodiferulic acid 8-O-4- and 5-5-derivatives were incubated with human fecal microbiota. The 8-*O*-4 derivative was shown to degrade temporarily to monomeric ferulic acid, which was then biotransformed into 3-(3′,4′-dihydroxyphenyl)propionic acid, 3′,4′-dihydroxyphenyl acetic acid, 3-phenylpropionic acid, and benzoic acid. An alternative pathway to benzoic acid is related to the metabolism of 3-(4′-hydroxy-3′-methoxyphenyl)pyruvic acid. Contrarily, the 5-5-diferulate derivatives were exposed only to demethylation and/or side-chain reductions [117].

#### 3.2.2. Stilbenes

Stilbenoids are very typical polyphenols in our diets, which mainly present in red grapes, wines, cranberries, strawberries, and peanuts [118,119]. Resveratrol and its derivatives are the most important dietary stilbenoids associated with many benefits for human health [120,121,122]. *trans*-Resveratrol is metabolized by human gut microbiota to dihydroresveratrol, 3,4′-dihydroxy-trans-stilbene and 3,4′-dihydroxybibenzyl (lunularin) *in vivo* [123]. *trans-*Piceid, the 3-*O*-β-d-glucoside of resveratrol, is metabolized to resveratrol, dihydropiceid and dihydroresveratrol by gut microbiota [124].

#### 3.2.3. Lignans

Lignans consist of pinoresinol, matairesinol, secoisolariciresinol, isolariciresinol, syringaresinol and lariciresinol diphenolic compounds with a 1,4-diarylbutane structure [125]. They are mostly found in fruits and vegetables, tea, cereal products, and coffee [126]. If consumed, they are potentially metabolized by microbial processes [2,127,128,129]. Lignans are considered phytoestrogens because of their estrogen agonist or antagonist properties [2]. The biological activity of lignans is associated with their activation into enterolactone and enterodiol, which are mammalian phytoestrogens [130,131,132]. The bioavailability of lignans is directly associated with intestinal bacterial metabolism [128]. These biotransformation reactions require demethylation and dehydroxylation [133].

### 3.3. Limitations for the Studies on Metabolism of Phenolics and Microbial/Colonic Metabolic Pathways

By reviewing the latest studies, it can be concluded that phenolic compounds are biotransformed by gut microbiota, generating intermediate and final related metabolites that could be present in the digesta in higher concentrations than their precursors. These phenolic metabolites do not share the same bioavailability and health effects with their parent compounds. In this context, there is an inherent limitation in the studies focused on analyzing the parent compounds in food to correctly estimate the phenomena occurring in the colon. Studies should focus on identifying whether the health benefits are associated with the parent compounds or their phenolic metabolites.

There are also many studies in the literature that observe the biotransformation of phenolic compounds in a food matrix, but studies using pure phenolic compounds are limited. Indeed, food has a complex matrix, and it is difficult to understand the phenolic–gut microbiota interactions directly, as some interactions with other food components might occur. It is important to understand the effect of gut microbiota on pure, individual phenolic compounds.

Another limitation is that it is not clear which intestinal bacteria have an effect on which phenolic compounds. Therefore, it is important to investigate the specific intestinal bacteria implicated in the metabolism of dietary polyphenols.

Phenolic compounds represent a large group, containing many sub-classes, as described. Although they have similar characteristics, there are important differences in their chemical structures, the number of functional groups, and the combination of different moieties, which lead to different functional activities. Despite the information obtained and published over recent years, microbial mechanisms of action on each phenolic compound and their metabolites remain unclear, and further research is needed to ascertain causal relationships.

## 4. Bioactivity and Bioavailability of Polyphenols are Affected by Gut Microbiota

Bioaccessibility is an important factor in determining bioavailability, and is a prerequisite for intestinal absorption. The process involves the release of compounds from an encapsulating food matrix that can be solubilized during digestion, and is potentially available for further absorption [7]. Polyphenols exert their functional influence dependent on intestinal absorption. Some polyphenols are found more distributed in nature but may not be bioavailable. The amount of bioaccessible phenolic compounds may differ significantly. Some phenolic compounds may be released and absorbed in small amounts, similar to carotenoids [134]. Some polyphenols, such as anthocyanins, might be degraded before reaching their site of absorption that results in bioaccessibility levels below 10% [135]. Therefore, a comprehensive understanding of the reactions that occur during digestion is essential to understand and estimate the bioaccessibility of these polyphenols.

Polyphenol structure is an important factor influencing bioavailability. Polyphenols that are most commonly found in dietary components are glycosides (e.g., flavonols, flavones, flavanones, isoflavones and anthocyanins). Less endemic oligomers (proanthocyanidins) are not absorbed by intestinal mucosa [136]. In the intestinal mucosa, only aglycones and some glucosides can be absorbed [137]. Thus, human digestive and microbial enzymes help to release native polyphenols from the food matrix, which is an essential mechanism for them to pass through the intestinal barrier [138,139]. The released aglycones and polyphenol monomers can be transported through passive diffusion and membrane permeases into enterohepatic circulation [136]. There they will be conjugated and returned to the small intestine, along with bile during passage into the liver, mainly by glucuronidation and sulphation reactions. Unabsorbed polyphenols in the small intestine are deconjugated by microbial glucuronidases and sulphatases in the colon, permitting the reuptake of aglycones [140,141]. In addition, intestinal microbiota are able to degrade aglycones and release simple aromatic compounds, including hydroxyphenylacetic acids from flavonols, hydroxyphenyl-propionic acids from flavones and flavanones and phenylvalerolactones and hydroxyphenylpropionic acids from flavanols. This renders them available for subsequent absorption and conjugation [139].

The enveloping food matrix that surrounds polyphenols influences the bioavailability of the molecules. Polyphenols that are integrated into a cellular structure can be released and absorbed in the intestinal mucosa during chewing and food digestion [140,141]. While some foods are consumed unprocessed, many food products require a degree of processing which may alter the bioavailability of phenolic compounds. For example, orange juice processing precipitates flavanones through interactions with pectins and other orange macromolecules, leading to lower bioavailability than the unprocessed phenolic compounds [142]. Furthermore, almond skin polyphenols have lower bioavailability after industrial bleaching [143].

Polyphenols also interact with other nutrients or ingredients that influence their bioavailability. Protein-phenolic interactions generally decrease bioavailability [144], as do associations with dietary fiber [145]. However, lipids seem to enhance the availability of phenolic compounds [146].

Two-way, reciprocal interactions of gut microbiota and phenolic compounds have an important impact on the bioavailability of phenolic compounds and human health. Gut microbiota plays an important role in harvesting nutrients from the diet [147]. Phenolic compounds that are not readily absorbed in the small intestine serve as growth substrates for the members of the gut microbial community, which in turn modify the bioavailability and nutritional properties of these compounds [147]. Phenol Explorer Database Release 2.0 [148] is a useful and capable resource, as it allows rapid retrieval of data on the biotransformations and pharmacokinetics of dietary polyphenols. Pharmacokinetic data on 380 metabolites identified in biofluids after the consumption of polyphenol-rich sources are presented in the database. These data have been extracted from 236 publications, and originate from 221 intervention studies in human subjects and experimental animals. This database is important for polyphenol scientists as bioactivities and health effects of polyphenols are dependent on the nature and concentrations of metabolites reaching the target tissues [148]. Higher bioavailability of phenolic compounds, and the resultant beneficial health effects as determined by the consumption of whole grains, vegetables and fruits, can be related to the end-products of microbial metabolism forming short-chain fatty acids such as butyrate, and phenolic acids such as protocatechuic acid [149,150,151,152].

In a very recent study by Esposito *et al.* (2015), the gastrointestinal distribution of black currant anthocyanins and their phenolic acid metabolites were examined in lean and diet-induced obese mice with healthy and antibiotic-disrupted microbiomes. Daily consumption of low-fat or high-fat diet supplemented with 1% black currant powdered extract (32% anthocyanins) for eight weeks reduced body weight gain and improved glucose metabolism only in mice with the intact gut microbiome. Administration of antibiotic cocktail resulted in a 16- to 25-fold increase (*p* < 0.001) in the anthocyanin content of feces, and cyanidin-based anthocyanins showed the largest increase in fecal content upon disruption of the gut microbiome, indicating their high susceptibility to microbial degradation in the gut. A three-fold increase in gallic acid over protocatechuic acid was observed in the jejunum of both intact and antibiotic-treated animals, suggesting that this effect was likely independent of gut microbiome status. As a result of this study, it can be concluded that the gut microbiome is necessary for the protective effect of black currant anthocyanins against obesity and the associated insulin resistance [153].

The bioavailability of red raspberry anthocyanins and ellagitannins was studied by Ludwig *et al.* (2015), considering their biotransformation by gut microbiota. They fed volunteers with red raspberries, containing ellagitannins and cyanidin-based anthocyanins, and analyzed metabolites appearing in plasma and urine by UHPLC-MS. They have indicated that metabolism of anthocyanins appears to start in the upper gastrointestinal tract, possibly pH-initiated, with 4′-hydroxyhippuric acid and ferulic acid derivatives circulating in plasma with a C_max_ of 1–1.5 h after consumption of raspberries. Furthermore, formation of these compounds was associated exclusively with the action of the colonic microflora. Ellagitannins pass from the small to the large intestine, where the colonic microbiota mediates the conversion to urolithins A and B. These compounds appeared in plasma and were excreted almost exclusively as sulfate and glucuronide metabolites. The urolithin metabolites persisted in the circulatory system and were excreted in urine for much longer periods of time than the anthocyanin metabolites, although their overall urinary recovery was lower, at 7.0% of intake. Events that originate in the proximal and distal gastrointestinal tract, and are succeeded by phase II metabolism, play an important role in the bioavailability of both anthocyanins and ellagitannins. Their metabolites, which appear in the circulatory system, are key to elucidating the mode, or modes, of action underlying the protective effects of these compounds on human health [154]. Similar results were obtained in previous bioavailability studies with anthocyanins and ellagitannins [155,156,157,158].

An important study explaining the effect of altered gut microbiota on the bioavailability of phenolics, performed by Dudonne *et al.* (2015), investigated the bioavailability of cranberry phenolics after oral administration of a cranberry extract (CE) to high-fat, high-sucrose (HFHS)-fed mice. Their work explored the possible modulation of gut microbiota composition following a co-supplementation with spores of *Bacillus subtilis* CU1 probiotic (CE/P). They extracted and characterized phenolic metabolites from plasma using µSPE-UHPLC-MS/MS, and a metagenomic analysis was performed on feces to assess gut bacterial composition. Twenty-two circulating metabolites were identified, mainly microbial degradation products of native cranberry phenolic compounds. Plasma concentration of three microbial metabolites was significantly increased with the CE/P co-treatment: *p*-coumaric acid, *m*-coumaric acid and *p*-hydroxybenzoic acid (+53%, +103% and +70%, respectively). They reported significant differences in the proportion of *Barnesiella* and *Oscillibacter* genera in CE/P-treated mice in comparison with control animals. This study pointed out that altered gut microbiota has a significant effect on the degradation and bioavailability of phenolic compounds in mice [159].

In contrast, it has been proposed that high concentrations of phenolic compounds could exert potential health benefits within the gastrointestinal tract [160]. Choy *et al.* (2013) studied the bioavailability of proanthocyanidins after ingestion of grape seed extract. Their findings indicate that ingested polymeric proanthocyanidins were present in the colon as the intact parent compounds and thus may contribute to the health of the gastrointestinal tract [161]. The same finding was reported previously, as in studies by He *et al.* (2005) that showed that high levels of anthocyanins in fecal content may play an important role in colonic health [162].

In summary, recent literature demonstrates that the mutual relationship between gut microbiota and phenolic compounds increases the bioavailability of phenolics and provides increased health benefits. Phenolic compounds can alter the gut microbiota community, resulting in a greater abundance of beneficial microbes, and a consequent increase in bioavailability. At the same time, phenolic compounds are biotransformed into their smaller metabolites by gut microbiota, which also contributes to increased bioavailability. The persistence of larger, intact phenolic compounds such as proanthocyanidins in the colon would allow these compounds to exert local beneficial biological actions, particularly on colonic epithelial cells, resulting in protective effects against inflammation-mediated diseases including colorectal cancer.

## 5. Polyphenols Modulate the Gut Microbiota Composition

Several phenolic compounds have been recognized as potential antimicrobial agents with bacteriostatic or bactericidal actions. In addition, they also inhibit bacterial infections of intestinal and urinary tract epithelia. Selma *et al.* (2009) reviewed the influence of human intestinal bacteria on health and the incidence of disease [42]. Gut health is mainly determined by the complex interactions between host and gastrointestinal microbiota. Beneficial bacteria such as *Bifidobaterium* spp. and *Lactobacillus* spp. have been observed to contribute to human health at different levels [163]. They enhance gut barrier function, stimulate the host immune system, prevent diarrhea or allergies, contribute to activation of provitamins, and modulate lipid metabolism [163,164]. However, there are other bacterial species associated with negative implications, such as *Clostridium difficile*, which has been associated with inflammatory bowel disease [165]. Therefore, it is of crucial importance to understand the inhibitory or stimulatory effect of phenolic compounds on beneficial or pathogenic bacteria, and their ratio in the gut. The influence of phenolic compounds on gut microbiota is provided in detail (Table 3).

### 5.1. Flavonoid-Type Phenolics

#### 5.1.1. Flavonols

Duda Chodak (2012) studied the impact of flavonols (*i.e.*, quercetin and rutin) on specific intestinal microbial species. In this study, six bacteria species (*Bacteroides galacturonicus*, *Lactobacillus* sp., *Enterococcus caccae*, *Bifidobacterium catenulatum*, *Ruminococcus gauvreauii*, and *Escherichia coli*) were inoculated with pure flavonols at final concentrations of 4, 20, and 50 μg/mL of quercetin and at 20, 100, and 250 μg/mL of rutin. Interestingly, quercetin showed a dose-dependent inhibitory effect on the growth of all analyzed bacterial species, whereas this effect was weaker for rutin [166].

Etxeberria *et al.* (2015) investigated whether quercetin administration could reverse the gut microbiota community attributable to a high-fat and high-carbohydrate diet and thereby impact health. Quercetin supplementation resulted in an altered composition of gut microbiota at different taxonomic levels, including the relative *Firmicutes*: *Bacteroidetes* ratio and inhibiting the growth of bacterial species associated with diet-induced obesity such as *Erysipelotrichaceae*, *Bacillus* spp., and *Eubacterium cylindroides* [167].

In an *in vitro* study conducted by Kawabata and colleagues (2013), *Bifidobacterium adolescentis*, a commensal often isolated from the human intestine was incubated with different flavonols (*i.e.*, quercetin, myricetin, galangin, kaempferol, and fisetin) to evaluate the effects of flavonols on the growth of *B. adolescentis*. Although galangin inhibited the growth of *B. adolescentis* by 30%–70% when co-cultured for 1–6 h, quercetin and fisetin showed no or little effect on the growth rate (20% inhibition at 6 h). Their study revealed that the tested flavonols, except for galangin, have no or weak anti-bacterial activity against *B. adolescentis* [168].

#### 5.1.2. Flavones and Flavanones

In a study by Parkar *et al.* (2008), many different polyphenols were demonstrated to influence the growth of human gut bacteria and their adhesion to enterocytes. Accordingly, naringenin promoted the growth of *Lactobacillus rhamnosus*, commensal *Escherichia coli*, along with two pathogens, *Staphylococcus aureus* and *Salmonella typhimurium*. In general, the Gram-positive enteropathogen *S. aureus* was the most sensitive to naringenin, while the Gram-negative pathogen *S. typhimurium* and the commensal organism *E. coli* were likely to be similar in their sensitivity to naringenin [169].

Moreover, Duda Chodak (2012) also studied the impact of naringenin and hesperetin on six bacteria species (*Bacteroides galacturonicus*, *Lactobacillus* sp., *Enterococcus caccae*, *Bifidobacterium catenulatum*, *Ruminococcus gauvreauii*, and *Escherichia coli*) and found out that they inhibited the growth of almost all analyzed bacteria (MIC ≥ 250 µg/mL) [166].

#### 5.1.3. Isoflavones

Isoflavones are transformed by gut microbiota, although there are few studies regarding the effect of isoflavone supplementation on gut microbiota composition. Parkar *et al.* (2008) investigated isoflavones (e.g., daidzein and genistein) on the growth of microbiota. As such, isoflavones induced a decrease in bacterial growth [168]. In another study by Clavel *et al.* (2005), postmenopausal women were supplemented with isoflavones (100 mg/day) for two months. As a result of the supplementation, it was observed that isoflavones changed predominant bacterial populations with enrichment of the *Clostridium coccoides*-*Eubacterium rectale* (Erec) cluster, *Faecalibacterium prasnutzii* subgroup, and *Lactobacillus*-*Enterococcus* group. The increased concentrations of the Erec cluster were proposed to be related with an intestinal metabolite from a daidzein named equol [170].

#### 5.1.4. Flavanols

Tzosunis *et al.* (2008) investigated the reciprocal metabolic interactions between gut microbiota and (−)-epicatechin and (+)-catechin using a pH-controlled, stirred, batch-culture fermentation system to model the distal colon. Interestingly, (+)-catechin influenced the growth of specific bacterial populations, including a statistically significant increase in the *Clostridium coccoides*–*Eubacterium rectale* group, *Bifidobacterium* spp. and *Escherichia coli*, as well as inhibiting the growth of the *Clostridium histolyticum* group. In contrast, (−)-epicatechin proved to be less active, only significantly increasing the *C. coccoides*–*Eubacterium* rectale group. These potential prebiotic effects for both (+)-catechin and (−)-epicatechin were most notable at a low concentration of 150 mg/L. As both (−)-epicatechin and (+)-catechin were converted to the same metabolites, the more dramatic change in the growth of distinct commensal populations produced by (+)-catechin incubation may be linked to the bacterial conversion of (+)-catechin to (−)-epicatechin. In summary, these data suggest that the consumption of flavanol-rich foods may support gut health through their ability to exert prebiotic actions [171].

In the study of Cueva *et al.* (2013), two flavan-3-ol fractions were used to investigate the *in vitro* fermentation with bacterial populations monitored by fluorescent *in situ* hybridization correlated with the appearance of phenolic metabolites. Both flavanol fractions promoted the growth of *Lactobacillus/Enterococcus* and decreased the *C. histolyticum* group during fermentation. Together, these data suggest that flavan-3-ol modulates microbiota composition and inherent catabolic activity, inducing changes that could affect the bioavailability and potential bioactivity of these compounds [172].

In an *in vivo* study done by Choy *et al.* (2014), six crossbred female pigs were fed with a diet containing grape seed extract for six days. DNA was extracted from pig fecal samples and the V3/V4 region of the 16S rRNA gene was sequenced using an Illumina MiSeq. The results indicated that the diet containing grape seed extract resulted in modulation of the gut microbiome, dramatically increasing *Lachnospiraceae*, *Clostridiales*, *Lactobacillus* and *Ruminococcaceae*. They have reported that the relationship between dietary proanthocyanidins and colon health may be attributed to the modulation of gut microbiota [173].

#### 5.1.5. Anthocyanins

Hidalgo *et al.* (2012) investigated the bacterial metabolism of malvidin-3-glucoside, gallic acid and a mixture of anthocyanins using an *in vitro* model of the human gut. The anthocyanins universally enhanced the growth of *Bifidobacterium* spp. and *Lactobacillus*-*Enterococcus* spp. significantly. This suggests that anthocyanins and their metabolites may positively select for beneficial members of the gut microbial community. Interestingly, malvidin-3-glucoside mixed with other anthocyanins exhibited a synergistic effect in promoting beneficial microbes [174].

Ultimately, there are very few *in vitro*, animal, and human intervention studies of anthocyanin interactions with gut microbiota. Thus, it is difficult to compare results and synthesize a conclusion, due in part to the varying techniques used to study microbiota, the different sources of anthocyanins, and study designs. Clearly, further research is necessary to clarify if anthocyanins have a direct or indirect effect on beneficial/pathogenic bacteria growth.

### 5.2. Nonflavonoid-Type Phenolics

#### 5.2.1. Phenolic Acids

In the study performed by Hidalgo *et al.* (2012), *in vitro* incubation of gallic acid in a fecal slurry reduced a group of potentially harmful bacteria such as *Clostridium histolyticum* without any negative effects on beneficial bacteria. In addition, it significantly reduced *Bacteroides* spp. growth and enhanced both the total bacterial number and the abundance of *Atopobium* spp. [174].

In another study, the influence of hydroxycinnamic acids such as caffeic acid, chlorogenic acid, *o*-coumaric acid, *p*-coumaric acid on the growth of a probiotic microbe (*Lactobacillus rhamnosus*), a commensal (*Escherichia coli*) and two pathogenic bacteria (*Staphylococcus aureus*, *Salmonella typhimurium*) was investigated. They compared the MIC values of all polyphenols tested and observed that flavonols, isoflavones and glycosides have a low antibacterial activity, while phenolic acids were found to be at an intermediate level. On the other hand, the flavanone and flavanol tested had high antibacterial activity [169].

#### 5.2.2. Hydrolyzable Tannins (Ellagitannins)

Ellagitannins are hydrolyzable tannins that are hydrolyzed *in vivo* to release ellagic acid. They produce urolithin when they are metabolized by the gut microbiota. The effect of these tannins on the growth of intestinal bacteria is inadequately characterized, and generally their antimicrobial potential has been assessed *in vitro*.

In an *in vitro* study conducted by Bialonska *et al.* (2009), the effect of a commercial pomegranate extract at a concentration of 0.01% as well as the effect of its main constituents (0.05%) on the growth of several species of human gut bacteria using a liquid culturing method was investigated. As a result of this study, it was observed that pomegranate byproducts and punicalagins inhibited the growth of pathogenic *Clostridia* and *Staphylococcus aureus*. Interestingly, probiotic *Lactobacilli* and *Bifidobacteria* were generally not affected by ellagitannins [175]. It is important to note that Bialonska *et al.* (2010) intended to prove whether this trend was maintained using a fermentation batch-culture system inoculated with fecal samples from healthy individuals, which better simulates conditions from the colonic region. In this experiment, pomegranate extract was able to produce an incremental increase on total bacterial number, enhancing the growth of *Bifidobacterium* spp., *Lactobacillus* and *Enterococcus* groups, while no effect was observed for the *C. histolyticum* group [176].

In the study of Larrosa *et al.* (2010), the ellagitanins of pomegranate and their main microbiota- derived metabolite urolithin A have also been identified to be responsible for changes in intestinal microbiota in rats with an increase of *Bifidobacterium* and *Lactobacillus* levels [177].

A different *in vivo* study, Li *et al.* (2015) had 20 healthy volunteers consume 1000 mg of pomegranate extract (POM) for four weeks. Changes in gut microbiota composition were monitored. Three distinct groups were established according to the urinary and fecal content of the POM metabolite urolithin A (UA). These groups were classified as (1) individuals with no baseline UA presence but induction of UA formation by POM extract consumption (*n* = 9); (2) baseline UA formation which was enhanced by POM extract consumption (*n* = 5); and (3) no baseline UA production, which was not inducible (*n* = 6). According to the results, *Actinobacteria* was increased and *Firmicutes* decreased significantly in individuals forming UA (producers). Additionally, *Verrucomicrobia* (*Akkermansia muciniphila*) was 33- and 47-fold higher in stool samples of UA producers compared to non-producers at baseline and after four weeks, respectively. In UA producers, the genera *Butyrivibrio, Enterobacter, Escherichia, Lactobacillus, Prevotella, Serratia* and *Veillonella* increased in abundance, and *Collinsella* decreased significantly at week 4 compared to the baseline. The consumption of POM extract resulted in the formation of its metabolites in some but not all participants. POM extract consumption may induce health benefits secondary to changes in the microbiota [178].

#### 5.2.3. Stilbenes

The antimicrobial effects of resveratrol (3,5,4′-trihydroxy-*trans*-stilbene) against several pathogenic and beneficial bacteria have been reported. In the *in vivo* study of Larrosa *et al.* (2009), rats were fed with 1 mg of resveratrol/kg/day (a human-equivalent dose) for 25 days, and in the last five days, 5% dextran sulfate sodium (DSS) was administered to induce colitis. Effects on colon tissue damage, gut microbiota, reactive oxygen species, inflammatory markers and nitric oxide production as well as gene expression profile with microarrays were evaluated. Resveratrol increased lactobacilli and bifidobacteria as well as diminished the increase of enterobacteria upon DSS treatment [179].

In another study of Qiao *et al.* (2014), mice were supplemented by resveratrol (200 mg per kg per day) and the changes in gut microbiota were monitored and quantified by fluorescence *in situ* hybridization and flow cytometry methods. Results of this study showed that resveratrol ameliorates the dysbiosis in the gut microbiota induced by the high-fat diet, specific effects including an increase in the *Bacteroidetes*-to-*Firmicutes* ratio, significant inhibition of the growth of *Enterococcus faecalis*, and increased growth of *Lactobacillus* and *Bifidobacterium* [180].

Etxeberria *et al.* (2015) determined whether *trans*-resveratrol administration could affect gut microbiota modulation produced by a high-fat sucrose (HFS) diet, thereby improving gut health. *Trans*-resveratrol supplementation significantly reduced the mean relative abundance of different *Clostridia* species such as *Clostridium aldenense* (−93.1%), *Clostridium hathewayi* (−73.2%), *Clostridium* sp. C9 (−76.3%) and *Clostridium* sp. MLG661 (−53.7%). This data is in opposition to the mean relative abundance of *Clostridium* sp. XB90 (266.6%) which was notably enhanced when compared to the HFS-diet-fed control rats. Additionally, the percentage of change in the mean relative abundance of *Gracilibacter thermotolerans* (−57.7%) and *Parabacteroides distasonis* (−77.4%) was negatively affected by *trans*-resveratrol in comparison to that detected in the HFS-diet-fed control group. Moreover, *trans*-resveratrol supplementation produced a statistically significant inhibition in the *Graciibacteraceae* family (−57.7%) compared to the HFS-diet-fed control rats. As a result of this study, it is stated that *trans*-resveratrol supplementation alone or in combination with quercetin almost modified the profile of gut bacteria, but instead acted at the intestinal level [167].

#### 5.2.4. Lignans

In the study of Niemi *et al.* (2013), a lignin-rich fraction originating from brewers’ spent grain did not suppress the conversion activity of gut microbiota in an *in vitro* colon metabolic model, nor did it inhibit the growth of beneficial gut bacteria, lactobacilli and bifidobacteria [180]. Moreover, some component in the protease-alkaline extracted fraction enabled the growth of bifidobacteria for a longer time than glucose [181].

**Table 3:** Influence of phenolic compounds in gut microbiota composition.

Polyphenol Type	Tested Bacteria	Growth (+)/Inhibitory (−) Effect	Type of Study	Methods Used	Duration	Doses	References
***IN VITRO* CELL CULTURE STUDIES**							
**Flavonols**							
Quercetin							
	*Bacteroides galacturonicus*	(−)	*In vitro*	Counting on culture medium	24 h	4, 20 or 50 μg/mL	[166]
	*Lactobacillus* sp.	(−)					
	*Enterococcus caccae*	(−)					
	*Bifidobacterium catenulatum*	(−)					
	*Ruminococcus gauvreauii*	(−)					
	*Escherichia coli*	(−)					
Rutin						20, 100 or 250 μg/mL	
	*Bacteroides galacturonicus,*	NS					
	*Lactobacillus* sp.	(+)					
	*Enterococcus caccae*	NS					
	*Bifidobacterium catenulatum*	(−)					
	*Ruminococcus gauvreauii*	NS					
	*Escherichia coli*	(−)					
**Flavonols**	*Bifidobacterium adolescentis*		*In vitro*	Counting on culture medium	24 h	flavonol (galangin,kaempferol, quercetin, myricetin, or fisetin dissolved in dimethylsulphoxide (DMSO); final 25 µM; final 0.1% DMSO	[168]
Galangin		(−)					
Kaempferol		NS					
Quercetin		NS					
Myricetin		NS					
Fisetin		NS					
**Isoflavones**		MIC (μg/mL)	*In vitro*	Minimum Inhibitory Concentration Assay (MIC)	1 h	Concentrations ranging from 62.5 to 1000 μg/mL	[170]
**Daidzein**	*Eschericia coli*	1000					
	*Staphylococcus aureus*	125					
	*Salmonella typhimirum*	1000					
	*Lactobacillus rhamnosus*	1000					
**Genistein**	*Eschericia coli*	1000					
	*Staphylococcus aureus*	125					
	*Salmonella typhimirum*	1000					
	*Lactobacillus rhamnosus*	1000					
**Flavanones**		MIC (μg/mL)	*In vitro*	Minimum Inhibitory Concentration Assay (MIC)	1 h	Concentrations ranging from 62.5 to 1000 μg/mL	[169]
**Naringenin**	*Eschericia coli*	125					
	*Staphylococcus aureus*	62.5					
	*Salmonella typhimirum*	125					
	*Lactobacillus rhamnosus*	125					
**Phenolic acids**		MIC (μg/mL)	*In vitro*	Minimum Inhibitory Concentration Assay (MIC)	1 h	Concentrations ranging from 62.5 to 1000 μg/mL	[169]
caffeic acid	*Eschericia coli*	500					
	*Staphylococcus aureus*	125					
	*Salmonella typhimirum*	500					
	*Lactobacillus rhamnosus*	≤250					
chlorogenic acid	*Eschericia coli*	1000					
	*Staphylococcus aureus*	125					
	*Salmonella typhimirum*	1000					
	*Lactobacillus rhamnosus*	≤250					
*o*-coumaric acid	*Eschericia coli*	250					
	*Staphylococcus aureus*	125					
	*Salmonella typhimirum*	250					
	*Lactobacillus rhamnosus*	250					
*p*-coumaric acid	*Eschericia coli*	500					
	*Staphylococcus aureus*	125					
	*Salmonella typhimirum*	500					
	*Lactobacillus rhamnosus*	500					
**Ellagitannins**	POMx		*In vitro*	Liquid culturing method	POMx (100 mL)	comercial extract of pomegranate at 0.01% as well as the effect of its main constituents (0.05%)	[175]
Extract of pomegranate (POMx) and its main constituents (punicalagins, punicalins, elagic acid, gallic acid)	*L. acidophilus*	(+)					
	*L. casei* ssp. *casei*	NS					
	*L. paracasei* ssp.	NS					
	*L. pentosus*	(+)					
	*L. rhamnosus*	(+)					
	*B. breve*	(+)					
	*B. infantis*	(+)					
	*B. longum*	NS					
	*B. bifidum*	(+)					
	*B. animalis* ssp. lactis	NS					
	*Bacteroides fragilis*	NS					
	*C. perfringens*	(−)					
	*Clostridium clostriidoforme*	NS					
	*C. ramosum*	(−)					
	*S. aureus*	(−)					
	Punicalagin						
	*L. acidophilus*	NS					
	*L. casei* ssp. *casei*	NS					
	*L. paracasei* ssp.	NS					
	*L. pentosus*	NS					
	*L. rhamnosus*	NS					
	*B. breve*	(+)					
	*B. infantis*	NS					
	*B. longum*	NS					
	*B. bifidum*	NS					
	*B. animalis* ssp. lactis	(+)					
	*Bacteroides fragilis*	NS					
	*C. perfringens*	(−)					
	*Clostridium clostriidoforme*	(−)					
	*C. ramosum*	(−)					
	*S. aureus*	(−)					
	*Punicalin*						
	*L. acidophilus*	NS					
	*L. casei* ssp. *casei*	NS					
	*L. paracasei* ssp.	NS					
	*L. pentosus*	NS					
	*L. rhamnosus*	NS					
	*B. breve*	NS					
	*B. infantis*	NS					
	*B. longum*	NS					
	*B. bifidum*	NS					
	*B. animalis* ssp. lactis	(+)					
	*Bacteroides fragilis*	NS					
	*C. perfringens*	NS					
	*Clostridium clostriidoforme*	NS					
	*C. ramosum*	NS					
	*S. aureus*	NS					
***IN VITRO* FAECAL MICROBIOTA STUDIES**							
Flavonols	*Erysipelotrichaceae*	(−)	*In vitro*	16S rDNA reads	6 weeks	30 mg/kg BW/day	[167]
Quercetin	*Ruminococcaceae*	NS					
	*Clostridiaceae*	NS					
	*Bacteroidaceae*	NS					
	*Lachnospiraceae*	NS					
	*Acidaminococcaceae*	NS					
	*Eubacteriaceae*	NS					
	*Prevotellaceae*	NS					
	*Acholeplasmataceae*	NS					
	*Lactobacillaceae*	NS					
	*Graciibacteraceae*	NS					
	*Clostridium aldenense*	NS					
	*Clostridium hathewayi*	NS					
	*Bacteroides vulgatus*	(+)					
	*Clostridium clariflavum*	(+)					
	*Clostridium methylpentosum*	NS					
	*Clostridium* sp. C9	NS					
	*Clostridium* sp. XB90	NS					
	*Clostridium* sp. MLG661	(+)					
	*Blautia stercoris*	NS					
	*Gracilibacter thermotolerans*	NS					
	*Parabacteroides distansonis*	NS					
	*Eubacterium cylindroides*	(−)					
	*Akkermansia muciniphila*	NS					
	*Bilophila wadsworthia*	NS					
	*Bacteroides* sp. dnLKV7	NS					
	*Barnesiella intestinihominis*	NS					
	*Bacteroides* sp. S-18	NS					
	*Bacteroides chinchillae*	NS					
	*Candidatus Prevotella conceptionensis*	NS					
**Flavanols**			**		10 h (150 mg/L)	150 mg/L and 1000 mg/L	[171]
(+)-catechin	*Bifidobacterium* spp.	(+)	**		17 h (1000 mg/L)	150 mg/L and 1000 mg/L	
	*Bacteroides* spp.	NS	**				
	*Lactobacillus/Enterococcus* spp.	NS	**				
	*Clostridium coccoides–Eubacterium rectale* group	(+)	**				
	*C. histolyticum* group	(−)	*In vitro*	Fluorescent *in situ* hybridization (FISH)			
	*Escherichia coli*	(+)	**				
(−)-epicatechin			**				
	*Bifidobacterium* spp.	NS	**				
	*Bacteroides* spp.	NS	**				
	*Lactobacillus/Enterococcus* spp.	NS	**				
	*Clostridium coccoides–Eubacterium rectale* group	(+)	**				
	*C. histolyticum* group	NS	**				
	*Escherichia coli*	NS	**				
**Flavan-3-ols**	*Lactobacillus/Enterococcus*	(+)	*In vitro*	Fluorescent *in situ* hybridization (FISH)	Samples were collected at 0, 5, 10, 24, 30 and 48 h of fermentation	600 mg/L	[172]
2 fractions of grape seed	*Clostridium histolyticum*	(−)	Human fecal microbiota				
**Anthocyanins**			*In vitro*	Fluorescent *in situ* hybridization (FISH)	0, 1, 2, 4, 5, 10, and 24 h	20 mg/L and 200 mg/L	[174]
Malvidin-3-glucoside	Total bacteria count	(+)	**				
	*Atopobium* spp.	(+)	**				
	*Bif idobacterium* spp.	(+)	**				
	*C. cocoides−Eubacterium rectale*	(+)	**				
	*Bacteroides* spp.	(−)	**				
	*Lactobacillus* spp.	(+)	**				
	*Clostridium histolyticum*	(−)	**				
**Stilbenes**	*Erysipelotrichaceae*	NS	*In vitro*	16S rDNA reads	6 weeks	15 mg/kg BW/day	[167]
**Trans-resveratrol**	*Ruminococcaceae*	NS	**				
	*Clostridiaceae*	NS	**				
	*Bacteroidaceae*	NS	**				
	*Lachnospiraceae*	NS	**				
	*Acidaminococcaceae*	NS	**				
	*Eubacteriaceae*	NS	**				
	*Prevotellaceae*	NS	**				
	*Acholeplasmataceae*	NS	**				
	*Lactobacillaceae*	NS	**				
	*Graciibacteraceae*	(−)	**				
	*Clostridium aldenense*	(−)	**				
	*Clostridium hathewayi*	(−)	**				
	*Bacteroides vulgatus*	NS	**				
	*Clostridium clariflavum*	NS	**				
	*Clostridium methylpentosum*	NS	**				
	*Clostridium* sp. C9	(−)	**				
	*Clostridium* sp. XB90	(+)	**				
	*Clostridium* sp. MLG661	(−)	**				
	*Blautia stercoris*	NS	**				
	*Gracilibacter thermotolerans*	(−)	**				
	*Parabacteroides distansonis*	(−)	**				
	*Eubacterium cylindroides*	NS	**				
	*Akkermansia muciniphila*	NS	**				
	*Bilophila wadsworthia*	NS	**				
	*Bacteroides* sp. dnLKV7	NS	**				
	*Barnesiella intestinihominis*	NS	**				
	*Bacteroides* sp. S-18	NS	**				
	*Bacteroides chinchillae*	NS	**				
	*Candidatus Prevotella conceptionensis*	NS	**				
**Phenolic acids**	*Total bacteria count*	(+)	*In vitro*	Fluorescent *in situ* hybridization (FISH)	0, 1, 2, 4, 5, 10, and 24 h	150 mg/L and 1000 mg/L	[174]
Gallic acid	*Atopobium* spp.	(+)	**				
	*Bif idobacterium* spp.	(+)	**				
	*C. cocoides−Eubacterium rectale*	(+)	**				
	*Bacteroides* spp*.*	(−)	**				
	*Lactobacillus* spp.	(+)	**				
	*Clostridium histolyticum*	(−)	**				
**Ellagitannins**	Total bacteria	(+)	*In vitro*	batch-culture fermentation system inoculated with fecal samples from healthy individuals, FISH	Samples collected at 0, 5, 10, 24 and 48 h	POMx (1.5 mL) and punicalagins (250 mg) were inoculated in stirring batch-culture vessels (one per treatment) containing faecal slurry (1:10, *w/v*).	[175]
pomegranate by-product (POMx)	*Bifidobacterium* spp.	(+)	**				
	*Lactobacillus* spp.	(+)	**				
	*Clostridium coccoides–Eubacterium* *rectale group C. histolyticum* group	(+)	**				
		NS	**				
**Lignans**	*Lactobacillus rhamnosus* VTT E-97800	(+)	*In vitro* colon model	Counting on culture medium	0, 2, 4, 6, 8, and 24 h	8 mL of fecal suspension, and a 16.7% (*w/v*) final concentration of fresh fecal matter	[179]
Lignins	*L. rhamnosus* VTT E-97948	(+)	**				
	*Lactobacillus paracasei* VTT E-97949	(+)	**				
	*Lactobacillus salivarius* VTT E-981006	(+)	**				
	*Bifidobacterium adolescentis* VTT E-981074, *Bifidobacterium breve* VTT E-981075, *Bifidobacterium longum* VTT E-96664	(+)	**				
	*Lactobacillus rhamnosus* VTT E-97800,	(+)	**				
	*L. rhamnosus* VTT E-97948	(+)	**				
***IN VIVO* STUDIES**							
**Isoflavones**	*Clostridium coccoides-Eubacterium rectale* cluster	(+)	*In vivo*	FISH and flow cytometry analyses	2 months (Fecal samples were collected on day 0, 30, and 60)	100 mg/day of isoflavones aglycon equivalents	[170]
	*Lactobacillus-Enterococcus* group*,*	(+)	**				
	*Faecalibacterium prausnitzii* subgroup*, Bifidobacterium* genus	(+)	**				
	*Clostridium coccoides-Eubacterium rectale* cluster	(+)	**				
**Condensed Tannins**	*Lachnospiraceae*	(+)	*In vivo*	culture-independent barcoded next generation sequencing	3 days normal diet	1% w/w Grape Seed Extract	[173]
Proanthocyanidins	*Clostridiales*	(+)	**		6 days treatment diet		
	*Lactobacillus*	(+)	**		3 days post treatment control-feeding		
	*Ruminococcaceae*	(+)	**		fecessamples taken daily		
**Stilbenes**			*In vivo*	Agar dilution method	25 days	1 mg/kg/day	[179]
Resveratrol	*Lactobacillus*	(+)	**				
	*Bifidobacterium*	(+)	**				
	*Enterobacteria*	Diminished the increase	**				
**Stilbenes**	*Bacteroidetes*-to-*Firmicutes* ratio,	(+)	*In vivo*	FISH and flow cytometry	12 weeks	200 mg/kg/day	[180]
Resveratrol	Enterococcus faecalis	(−)	**				
	Lactobacillus	(+)	**				
	Bifidobacterium	(+)	**				
**Ellagitannins**	*Lactobacilli*	(+)	*In vivo*	Agar dilution method with fecal microbiota of rats	Samples collected at days 0, 10, 20	250 mg/kg/day	[177]
Pomegranate ellagitannins and their microbiota-derived metabolite urolithin A	*Bifidobacterium*	(+)	**				
**Ellagitannins**	*Actinobacteria*	(+)	*In vivo*	FISH and flow cytometry	4 weeks	1000 mg POM extract	[178]
Pomegranate (POM) ellagitannins	*Firmicutes*	(−)	**				
	*Verrucomicrobia (Akkermansia muciniphila)*	(+)	**				
	*Butyrivibrio*	(+)	**				
	*Enterobacter*	(+)	**				
	*Eschericia*	(+)	**				
	*Lactobacillus*	(+)	**				
	*Prevotella*	(+)	**				
	*Serratia*	(+)	**				
	*Veillonella*	(+)	**				
	*Collinsella*	(−)	**				

NS: no significant difference, (+): increase, (−): decrease.

### 5.3. Limitations for the Studies on Gut Microbiota Composition Modulation by Polyphenols

The effect of phenolic compounds on gut microbiota modulation has gained much attention in recent years, but the influence of polyphenols on specific gut bacteria is still not clear. One of the main limitations in previous studies is that most phenolic fractions and pure phenolic compounds have been analyzed without considering the bioavailability and the chemistry of phenolic compounds in the colon.

Another limitation is that the information obtained from *in vitro* studies about the role of individual phenolic compounds on gut microbiota cannot be directly extrapolated to what occurs in the physiological context of the gut ecosystem. Human and animal intervention studies involve very high doses of individual phenolic compounds, or high amounts of foods rich in phenolic content, neither of which represents the regular diet. Therefore, there is a lack of adequate *in vivo* studies.

Human intervention studies provide the best models for studying the effect of phenolic compounds on gut microbiota modulation. However, *in vivo* human intervention studies hold inevitable practical and ethical limitations. Few studies have observed the *in vivo* impact of phenolic compounds on the human gut microbiota [170,173,177,178,179,180]. Of those performed, most were focused on single polyphenol molecules and selected bacterial populations. Further *in vivo* research is needed to understand the effect of phenolic compounds on gut microbiota.

There may be a wide variability in response to phenolic compounds according to the differences in gut microbiota composition. It is difficult to understand the relationship between phenolic compounds and gut microbiota according to these inter-individual differences, especially if there are different diet-microbiota relations. Future studies should be done considering the inter-individual differences in gut microbiota while studying effect of phenolic compounds on gut microbiota modulation from the immunological point of view.

After a thorough review of the latest studies on the effect of phenolic compounds on gut microbiota, it can be stated that phenolic compounds and their metabolites contribute to beneficial gastrointestinal health effects by modulating gut microbial balance with the simultaneous inhibition of pathogens and stimulation of beneficial bacteria. These latest studies indicate that the concept of prebiotics is not limited to non-digestible carbohydrates, but also applies to phenolic compounds that have the ability to show prebiotic action [9,48,168,171,177]. Therefore, the regular consumption of phenolic compound–rich foods in a diet may beneficially balance the gut microbiota and exert beneficial health effects.

## 6. Conclusions

The interactions between dietary components, especially phenolic compounds and gut microbiota, have gained much attention due to their relevance to bioavailability and human health. Even though there are a number of important studies on this topic, the data presented in these papers are generally focused on the one-way relationship between phenolics and gut microbiota, or on bioavailability. On the other hand, this review presents an overview of the reciprocal relationship of all the subclasses of phenolic compounds and gut microbiota, as well as the relevance of these interactions to bioavailability and human health.

When the studies are investigated, it is clear that there are some limitations and problems with respect to the present applications and results. For example, the studies presented in the literature largely investigated the biotransformation of phenolic compounds in a food matrix. However, studies using pure phenolic compounds are lacking, making it difficult to understand the exact mechanism of each individual compound. On the other hand, phenolic compounds are biotransformed by gut microbiota generating phenolic metabolites which may have different bioavailability in comparison to their parent compounds. Moreover, there are some limitations for the *in vivo* studies including ethical and economical issues, their complicated nature during application, and difficulties in translating the *in vitro* data into *in vivo* conditions.

Apart from the concerns noted above, studies focusing on the effect of two-way mutual interactions between gut microbiota and phenolic compounds are critical, as these results may lead to new information with respect to health. Indeed, future studies are still required to understand this complicated mechanism. The fact that phenolic compounds may balance the gut microbiota and contribute to gastrointestinal health, and may indeed exert prebiotic activity, makes it clear that it is important to clarify these mechanisms specifically to understand which bacteria will affect which phenolic compounds.

## Abbreviations

3,4-DHPPA: 3,4-dihydroxyphenylpropionic acid
3-HPPA: 3-hydroxyphenylpropionic acid
4-HPPA: 4-hydroxyphenylpropionic acid
3-HBA: 3-hydroxybenzoic acid
4-HBA: 4-hydroxybenzoic acid
3,4-DHPPA: 3,4-dihydroxyphenylacetic acid
3-HPAA: 3-hydroxyphenylacetic acid
4-HPAA: 4-hydroxyphenylacetic acid
FISH: fluorescent *in situ* hybridisation
